# 

**Published:** 1892-02

**Authors:** 


CONTENTS.

Vol. 39. No. 2.

| | PAGE. |
|---|---|
| Special | 25 |
| The Coming Woman — Her Health | 25 |
| Talks with Dr. Mandeville | 27 |
| A Curious War Incident | 30 |
| A Ghostly Warning | 32 |
| Personal Beauty | 34 |
| Antics of Watches | 35 |
| National Habit of Drinking | 36 |
| Eye Troubles | 37 |
| Catarrh | 37 |
| Simulation of Death in the East | 38 |
| Proportions of the Human Figure | 38 |
| The Sky | 39 |
| Luck in Old Shoes | 39 |
| Self-Supporting | 40 |
| The Ear | 41 |
| Mythical Snakes | 41 |
| Some Curious Legal Nuisances | 42 |
| New Remedy for Snake Bites and Rabies | 43 |
| Miscellaneous | 43 |
| Literary | 47 |
| Bread and Meat | 48 |

INDEX TO ADVERTISEMENTS OF HALF A PAGE AND OVER.

| | PAGE. |
|---|---|
| A Christmas Gift | I |
| Cornish & Co | II |
| Bovinine | IV |
| Dr. J. C. Ayer & Co | V |
| Herba Vita | VIII |
| Walter Baker & Co | IX |
| Magee Emulsion Co | X |
| Rochester Lamp Co | X |
| Royal Baking Powder | XI |
| Buffalo Lithia Water | XI |

